# High dose haemodialysis and haemodiafiltration parameters and the relationship with advanced vascular calcification

**DOI:** 10.1186/s12882-020-01738-4

**Published:** 2020-03-06

**Authors:** Sung Keun Park, Won Joong Kim, Hyun Jin Kim, Hae Won Kim, Beom Kim, Hong Joo Lee, So-Young Lee, Yu Ho Lee, Dong-Jin Kim, Kyung-Hwan Jeong, Ju-Young Moon, Sang-Ho Lee, Shin Young Ahn, Gang Jee Ko, Jae-Hong Ryoo, Dong-Young Lee

**Affiliations:** 1 Center for Cohort Studies, Total Healthcare Center, Kangbuk Samsung Hospital, Sungkyunkwan University School of Medicine, Seoul, Republic of Korea; 2 Department of Internal Medicine, Hansol hospital, Seoul, Republic of Korea; 3 Department of Internal Medicine, Veterans Healthcare Service Medical Center, Seoul, Republic of Korea; 4 Department of Nephrology, Seoul Red Cross Hospital, Seoul, Republic of Korea; 5 Division of Nephrology, Department of Internal Medicine, CHA Bundang Medical Center, CHA University, Seongnam, Republic of Korea; 6 Department of Genetic Engineering, College of Life Science and Graduate School of Biotechnology, Kyung Hee University Global Campus, Yongin, Republic of Korea; 7 Division of Nephrology, Department of Internal Medicine, Kyung Hee University, Seoul, Republic of Korea; 8 Department of Internal Medicine, College of Medicine, Korea University, Seoul, Republic of Korea; 9 Departments of Occupational and Environmental Medicine, School of Medicine, Kyung Hee University, Seoul, Republic of Korea

**Keywords:** End-stage renal disease, Haemodialysis, Vascular calcification, Kt/V

## Abstract

**Background:**

Vascular calcification (VC) is a risk factor for cardiovascular disease in end-stage renal disease (ESRD) patients undergoing maintenance haemodialysis (MHD). However, evidence is still insufficient about the association between dialysis parameters and VC. Thus, this study was to evaluate association of dialysis parameters with VC.

**Methods:**

We enrolled 297 ESRD patients undergoing MHD at six distinct centers in Korea. Study participants were categorized into 3 groups by the scoring system of abdominal aortic calcification based on lateral lumbar radiography (no VC group: 0, mild VC group: 1–7 and advanced VC group: 8–24). We compared the features of dialysis parameters according to the severity of VC. Multivariate logistic regression analysis was used to calculate adjusted odd ratios (ORs) and 95% confidence interval (CI) for mild and advanced VC in each haemodialysis parameter (adjusted OR [95% CI]).

**Results:**

Pooled Kt/V (spKt/V), equilibrated Kt/V (eKt/V), standard Kt/V (stdKt/V) and the proportion of haemodiafiltration were increased along with the severity of VC. Multivariate regression analysis indicated that advanced VC was positively associated with spKt/V (5.27 [1.51–18.41]), eKt/V (6.16 [1.45–26.10]), stdKt/V (10.67 [1.74–65.52]) and haemodiafiltration (3.27 [1.74 to 6.16]).

**Conclusion:**

High dose dialysis and haemodiafiltration were significantly associated with advanced VC.

## Background

The prevalence of chronic kidney disease (CKD) is increasing worldwide, especially with the increasing prevalence of non-communicable diseases like diabetes mellitus (DM), hypertension and obesity [1]. CKD is a clinical manifestation of age-related decline of renal function. The increasing prevalence of CKD indicates that a considerable number of CKD patients may ultimately progress to end-stage renal disease (ESRD), depending on renal replacement therapy.

Haemodialysis is the most common method of renal replacement therapy in ESRD patients. ESRD patients that undergo haemodialysis have a 20–30-fold increased risk of cardiovascular mortality compared with an age-matched population [2], which represents the majority of all-cause mortality in ESRD patients. It is known that traditional cardiovascular risk factors like dyslipidaemia, hypertension, smoking, DM, obesity and advanced age contribute to pathological mechanisms of cardiovascular disease (CVD) in ESRD patients on haemodialysis [3].

Vascular calcification (VC) is highly prevalent in ESRD patients and is an independent predictor for cardiovascular (CV) morbidity and mortality. VC develops decades earlier in ESRD patients than in the general population [4], and dialysis accelerates the progression of VC [2]. VC is the consequence of the complex interactions between genetic, environmental, and vascular factors, which ultimately lead to the deposition of calcium in the vasculature [5]*.* Additionally, it is believed that clinical symptoms associated with dialysis and dialysis parameters affect the pathogenesis of VC.

Considering that VC is an independent predictor of CV prognosis in ESRD patients undergoing haemodialysis [6, 7], investigating factors related to VC may be helpful for reducing the risk of CVD in ESRD patients. However, data is on the association of clinical and dialysis parameters with VC is currently limited. Moreover, given that Asians are more predisposed to CVD, even at given metabolic conditions [8, 9], it will be important to identify the risk factors for VC in Asians.

In Korean ESRD patients receiving maintenance haemodialysis (MHD), we conducted a cross-sectional study to examine the clinical characteristics and dialysis parameters, according to the severity of VC. We also investigated factors that are associated with advanced VC.

## Methods

### Study subjects

Study subjects were recruited from a cohort of ESRD patients that were receiving MHD from six hospitals in Korea. The enrollment of study subjects was performed from June 2016 to June 2017. Cohorts were designed to assess the sociodemographic characteristics, underlying disease, nutritional status, exercise function, clinical exams, imaging findings and cardiologic work-up in ESRD patients receiving MHD. Through these assessments, the cohort study was aimed at identifying the risk factors for morbidity and mortality of major illnesses including cardiovascular complications.

The inclusion criteria of study subjects were as follows: receiving MHD at least 3 times a week, age ≥ 18 years and undergoing dialysis for more than 3 months. The exclusion criteria were as follows: the presence or history of malignant neoplasm, the presence or history of bone marrow disease, and life expectancy less than 6 months. The number of study participants who initially fulfilled the inclusion criteria was 411, and then 114 subjects were further excluded due to missing lumbar-spine lateral radiography data or withdrawal of consent. Finally, a total number of 297 subjects was enrolled in the study. All subjects voluntarily participated in the study, and informed consent was obtained in all cases. Ethics approvals for the study protocol and analysis of the data were obtained from the Institutional Review Board of Veterans Healthcare Service Medical Centre.

### Study data

Study data consisted of haemodialysis parameters and clinical parameters, including medical history, anthropometric measurements, biochemical analyses and hand grip strength. All blood exams were conducted right before haemodialysis and were performed in each hospital where study participants were receiving MHD. We evaluated past and current disease-like DM, hypertension, coronary artery disease (CAD) and cerebrovascular disease. Investigation of medication history included phosphate binder, vitamin D, statin, oral anticoagulation, iron, antihypertensive medication and erythropoiesis stimulating agents. Nutritional status was assessed by measuring the mid-arm mass circumference (MAMC) and performed by trained experts. Hand grip strength test was performed using a dynamometer (Fabrication Enterprises Inc., NY, USA), which was gripped with 90^°^ flexion of the forearm. Strength assessment with the gripping dynamometer was measured three times and recorded in kilograms by trained nurses [10].

### Vascular calcification assessment

**Fig. 1:**
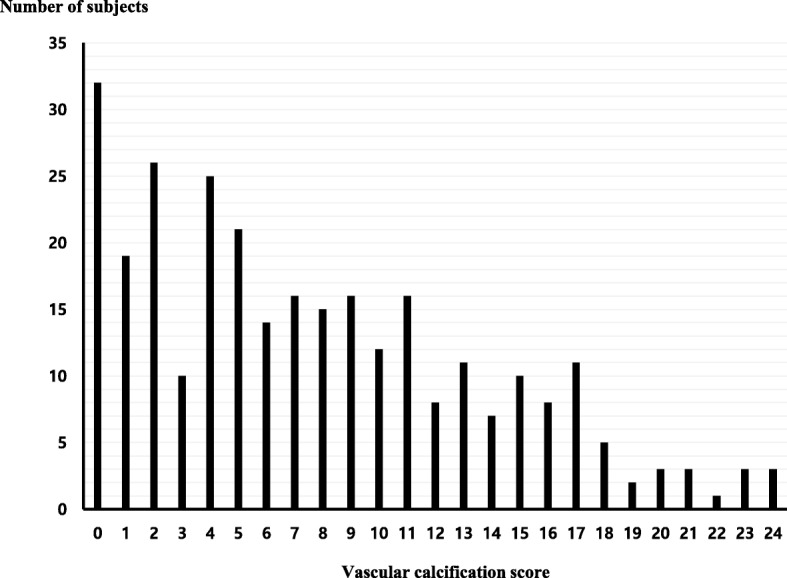
Distribution of vascular calcification score in study participants

A scoring system for abdominal aortic calcification based on lateral lumbar radiography was used to assess the severity of VC. The detailed methods of the scoring system for abdominal aortic calcification were described in a previous study [11]. The scores were calculated by the composite score for anterior–posterior severity (assigned here as the abdominal aortic calcification) where the scores of individual aortic segments both for the anterior and posterior walls were summed (maximum score 24). Abdominal aortic calcification from the lateral lumbar radiography was scored by two medical staff members who did not know the clinical state of patients. Inter-observer agreement was 91%. Figure 1 indicates the distribution of VC scores in study subjects. The median abdominal aortic calcification score was 7, which was used to define the severity of VC as follows: no VC (score: 0), mild VC (score: 1–7) and advanced VC (score: 8–24).

### Statistical analysis

Study subjects were classified into one of three groups with no VC, mild VC and advanced VC. Data are presented as means ± standard deviation for continuous variables and as proportions for categorical variables. Differences of clinical and dialysis parameters among the three groups were compared using the one-way analysis of variance test, the Kruskall-Wallis test for continuous variables and chi-square test for categorical variables. A Pearson product-moment correlation coefficient was calculated to analyse the correlation of the VC score with the values of each parameter.

Logistic regression analysis was used to calculate the crude and multivariate-adjusted odd ratio (OR) and the associated 95% confidence interval (CI) of each parameter for the advanced VC (adjusted OR [95% CI]). The adjusting covariates were age, sex, DM, dry body weight and mode of dialysis in model 1, and use of warfarin, dialysate calcium, and serum phosphate in model 2.

All statistical analyses were performed using SPSS Version 20 (IBM, Chicago, IL), and a *p* value < 0.05 was considered statistically significant in all analyses.

## Results

### Clinical and haemodialysis parameters

Clinical and haemodialysis parameters of study subjects across the severity of VC are presented in Table 1. Statistically significant differences were observed regarding age, DM, CAD, cerebrovascular disease, Charlson’s comorbidity index, sodium, chloride, corrected calcium, uric acid, total CO_2_ and hand grip strength. While the advanced VC group had relatively increased levels of age, DM, CAD, DM, CAD, cerebrovascular disease, Charlson’s comorbidity index, and uric acid, they had the relatively lower levels of serum sodium, chloride and hand grip strength test values. The no VC group had a higher BMI level (25.4 ± 5.1 Kg/m^2^) compared with the mild VC group (22.7 ± 3.3 Kg/m^2^) and the advanced VC group (22.9 ± 3.9 Kg/m^2^).

**Table 1:** Clinical and hemodialysis parameters of study patients

	All (*n* = 297)	No VC (*n* = 32)	Mild VC (*n* = 131)	Advanced VC (*n* = 134)	*P* value
Age (years)	62.7 ± 12.8	52.5 ± 14.6	61.2 ± 12.5	66.5 ± 10.9	0.000
Body mass index, (Kg/m^2^)	23.1 ± 3.9	25.4 ± 5.1	22.7 ± 3.3	22.9 ± 3.9	0.001
Male gender	210 (70.7%)	0	113 (53.8%)	97 (46.2%)	0.609
Comorbidities					
Diabetes	56.6	15 (46.9%)	65 (49.6%)	88 (65.7%)	0.016
Hypertension	87.2	29 (90.6%)	113 (86.3%)	117 (87.3%)	0.802
CAD	20.3	3 (9.4%)	17 (13.0%)	39 (29.1%)	0.001
CVD	24.2	2 (6.3%)	28 (21.4%)	41 (30.6%)	0.010
Charlson’s comorbidity index	4.1 ± 1.5	3.3 ± 1.3	4.0 ± 1.7	4.5 ± 1.3	0.000
Dialysis duration (months)	81.5 ± 73.5	51.4 ± 29.9	73.5 ± 66.0	93.3 ± 79.8	0.010
Dialysis mode					0.000
Haemodilaysis	220 (74.1%)	26 (81.3%)	110 (84.0%)	84 (62.7%)	
Haemodiafiltration	77 (25.9%)	6 (18.8%)	21 (16.0%)	50 (37.3%)	
Single pool Kt/V	1.55 ± 0.29	1.39 ± 0.30	1.51 ± 0.29	1.64 ± 0.27	0.000
Equilibrated Kt/V	1.35 ± 0.25	1.21 ± 0.25	1.31 ± 0.25	1.42 ± 0.23	0.000
Standard Kt/V	2.20 ± 0.20	2.08 ± 0.21	2.17 ± 0.20	2.26 ± 0.18	0.000
Urea reduction ratio (%)	72.6 ± 6.9	68.6 ± 7.2	71.6 ± 6.9	74.5 ± 6.2	0.000
Blood flow rate (ml/min)	265.5 ± 23.3	263.4 ± 15.6	260.8 ± 22.8	270.4 ± 24.3	0.003
Cinacalcet administration	21 (7.1%)	2 (6.3%)	6 (4.6%)	13 (9.7%)	0.214
Duration of HD (hours)	3.93 ± 0.19	3.95 ± 0.22	3.93 ± 0.17	3.94 ± 0.19	0.891
Pre-dialysis systolic BP (mmHg)	143.6 ± 19.8	143.8 ± 23.5	142.3 ± 21.3	144.8 ± 17.4	0.591
Pre-dialysis diastolic BP (mmHg)	73.3 ± 13.0	75.4 ± 11.4	73.2 ± 13.0	72.9 ± 13.4	0.606
Average UF per session (Kg)	2.2 ± 1.1	2.2 ± 1.3	2.2 ± 1.1	2.2 ± 0.9	0.930
Dry body weight (Kg)	62.8 ± 12.6	71.9 ± 18.3	62.2 ± 11.2	61.2 ± 11.8	0.000
Dialysate sodium (mEq/L)	137.0 ± 2.5	137.5 ± 2.5	137.4 ± 2.5	136.6 ± 2.3	0.012
Dialysate calcium (mEq/L)	2.8 ± 0.4	2.7 ± 0.4	2.8 ± 0.4	2.8 ± 0.4	0.415
Sodium (mmol/L)	137.6 ± 3.4	138.1 ± 3.0	138.4 ± 3.0	136.6 ± 3.7	0.000
Chloride (mmol/L)	99.2 ± 4.9	100.6 ± 3.7	100.3 ± 4.2	97.7 ± 4.9	0.000
Potassium (mmol/L)	4.8 ± 0.7	4.7 ± 0.8	4.7 ± 0.7	4.9 ± 0.8	0.169
Corrected Calcium (mg/dL)	8.6 ± 0.8	8.3 ± 0.7	8.5 ± 0.8	8.7 ± 0.8	0.009
Phosphate (mg/dL)	4.8 ± 1.4	4.8 ± 1.2	4.8 ± 1.6	4.7 ± 1.2	0.830
Total CO_2_ (mmol/L)	23.9 ± 3.1	23.7 ± 3.0	23.2 ± 3.4	24.5 ± 2.7	0.003
Serum albumin (mg/dL)	3.8 ± 0.4	3.8 ± 0.4	3.8 ± 0.3	3.7 ± 0.4	0.108
MAMC (cm)	22.9 ± 4.9	23.1 ± 6.9	23.0 ± 5.1	22.8 ± 4.6	0.941
nPCR (g/Kg/day)	1.0 ± 0.2	1.03 ± 0.20	1.03 ± 0.23	1.02 ± 0.25	0.918
Intact PTH (pg/mL)	269.7 ± 208.0	275.4 ± 276.6	258.6 ± 184.4	279.2 ± 212.2	0.715
Bone specific ALP (μg/L)	18.9 ± 10.9	14.8 ± 7.9	20.6 ± 12.6	18.3 ± 9.7	0.049
25(OH) Vitamin D (ng/mL)	16.4 ± 10.1	15.6 ± 8.7	16.2 ± 10.0	16.8 ± 10.6	0.807
hsCRP (mg/L)	7.0 ± 13.3	11.7 ± 20.3	7.2 ± 14.1	5.8 ± 9.7	0.081
Average hand grip strength test (Kg)	23.0 ± 10.3	29.0 ± 11.1	24.7 ± 9.9	19.3 ± 9.1	0.000
Vitamin D use	79 (26.6)	13 (40.6)	36 (27.5%)	30 (22.4%)	0.106
Statin use	143 (48.1%)	12 (37.5%)	61 (46.6%)	70 (52.2%)	0.289
Warfarin use	7 (2.4%)	0	1 (0.8%)	6 (4.5%)	0.089

Data are expressed as mean ± SD for continuous variables

*VC* Vascular calcification, *CADL* Coronary artery disease, *CVD* Cerebrovascular disease, *UF* Ultrafiltration, *MAMC* Mid-arm muscle circumference, *nPCR* Normalized protein catabolic rate, *SGA* Subject global assessment, *PTH* Parathyroid hormone, *ALP* Alkaline phosphatase, *CRP* C-reactive protein

While HD was more commonly used in the no VC group and mild VC group, HDF was more commonly used in the advanced VC group. Haemodialysis parameters, dialysis duration, single pool Kt/V (spKt/V), equilibrated Kt/V (eKt/V), standard Kt/V (stdKt/V) and urea reduction ratio (URR) tended to increase proportionally to the severity of VC, and post-dialysis DBP, dry body weight and dialysate sodium tended to decrease with the severity of VC.

Table 2 indicates the correlation analysis of clinical and haemodialysis parameters with advanced VC. A positive correlation was observed in age, blood flow rate, HD vintage, spKt/V, eKt/V, stdKt/V, URR, Charlson’s comorbidity index, potassium, uric acid, and corrected calcium, whereas a negative correlation was observed in dry body weight, dialysate sodium, hand grip test, albumin and chloride.

**Table 2:** Correlation analysis between abdominal aorta calcification and other factors

	R	*P* value
Age	0.325	0.000
Charlson’s comorbidity index	0.247	0.000
HD vintage	0.176	0.002
Single pool Kt/V	0.310	0.000
Equilibrated Kt/V	0.306	0.000
Standard Kt/V	0.317	0.000
Urea reduction rate	0.305	0.000
Blood flow rate	0.208	0.000
Dry body weight	−0.143	0.013
Dialysate sodium	−0.215	0.000
Sodium	−0.188	0001
Chloride	−0.290	0.000
Potassium	0.121	0.037
Corrected calcium	0.210	0.000
Total CO_2_	0.241	0.000
Serum albumin	−0.159	0.006
Hand grip test	−0.351	0.000

R Partial correlation coefficients, spKt/V single pool Kt/V, eKt/V equilibrated Kt/V, stdKt/V standard Kt/V, UF ultrafiltration, nPCR normalized protein catabolic rate

### Association of parameters with advanced VC

The unadjusted and adjusted ORs for advanced VC in clinical and dialysis parameters are presented in Table 3. Advanced VC was positively associated with CAD (2.78 [1.41–5.51]), spKt/V (5.27 [1.51–18.41]), eKt/V (6.16 [1.45–26.10]), standard Kt/V (10.67 [1.74–65.52]), HDF (3.27 [1.74–6.16]), corrected Ca (1.70 [1.17–2.46]), Charlson’s comorbidity index (1.86 [1.42–2.21]) and dialysis duration (2.34 [1.67–2.94]). Sodium (0.88 [0.81–0.96]) and chloride (0.90 [0.83–0.96]) levels were inversely associated with advanced VC. Even after incorporating Charlson’s comorbidity index and the duration of dialysis into adjusting covariates, advanced VC was significantly associated with spKt/V (3.09 [2.47–3.51]), eKt/V (3.42 [2.61–4.14]), standard Kt/V (6.21 [4.93–7.63]) and HDF (2.35 [1.94–2.94]) (Supplementary Table 1).

**Table 3:** Odds ratio (95% CI) for advanced vascular calcification in each haemodialysis parameters

	Unadjusted model	Model 1^a^	Model 2^b^
	OR (95% CI)	OR (95% CI)	OR (95% CI)
DM	1.99 (1.24–3.17)	1.88 (1.10–3.17)	1.94 (1.14–3.29)
CAD	2.93 (1.61–5.34)	2.64 (1.36–5.14)	2.78 (1.41–5.51)
Single pool Kt/V	6.5 (2.80–15.3)	5.32 (1.56–18.14)	5.27 (1.51–18.41)
Equilibrated Kt/V	8.8 (3.20–24.20)	6.33 (1.53–26.17)	6.16 (1.45–26.10)
Standard Kt/V	18.2 (4.90–68.20)	10.91 (1.83–65.21)	10.67 (1.74–65.52)
HDF (reference HD)	2.66 (1.51–4.68)	3.28 (1.76–6.12)	3.27 (1.74–6.16)
Sodium	0.85 (0.79–0.92)	0.88 (0.81–0.96)	0.88 (0.81–0.96)
Chloride	0.87 (0.83–0.93)	0.89 (0.83–0.95)	0.90 (0.83–0.96)
Corrected Ca	1.73 (1.25–2.40)	1.60 (1.12–2.27)	1.70 (1.17–2.46)
Charlsons’ comorbidity index	2.71 (2.24–3.31)	2.34 (1.86–2.91)	1.86 (1.42–2.21)
Dialysis duration (months)	3.34 (2.87–3.94)	2.81 (2.31–3.35)	2.34 (1.67–2.94)

*OR* Odds ratio, *CI* Confidence interval, *DM* Diabetes mellitus, *CAD* Coronary artery disease, *HD, HDF* Haemodiafiltration, *Ca* Calcium

^a^Adjusted for age, sex, DM, CAD, and dry BW

^b^Adjusted for use of warfarin, dialysate calcium, and serum phosphate

## Discussion

CVD is a leading cause of mortality in ESRD patients undergoing haemodialysis. VC is regarded as a risk factor for CVD. We investigated the association between clinical and haemodialysis parameters with VC in a multicentre observational study.

In our analysis, advanced VC was positively associated with DM, CAD, Kt/V categories, HDF, Ca, Charlson’s comorbidity index and duration of dialysis. It has already been established that DM, CAD and high Ca values are associated with VC. However, there is little information about the association between the dialysis dose and VC. The Kt/V categories, blood flow rate and URR are indicators of dialysis dose and used to assess the adequacy of haemodialysis. Our results showed that spKt/V, eKt/V and stdKt/V were positively associated with advanced VC. These findings suggest that high dose dialysis is potentially facilitative to the progression of VC. To date, there has been wide debate over the influence of dialysis dose on the prognosis in ESRD patients.

In a randomized trial from The National Cooperative Dialysis Study, the potential benefit of increased dialysis dose was suggested by a finding that more efficient removal of urea appeared to lead to decreased morbidity [12]. Some observational studies also showed that increased dialysis doses above guidelines were associated with improvements in all-cause mortality [13, 14]. However, several reports are in agreement with our findings, which do not support the potential benefit of high dose dialysis. A randomized clinical trial that enrolled 1846 patients demonstrated that high dose dialysis with URR values of 75.2 ± 2.5% and spKt/V of 1.71 ± 0.11 did not present any clinical benefits regarding all-cause mortality and hospitalization, compared with standard dose dialysis with URR values of 66.3 ± 2.5% and spKt/V of 1.32 ± 0.09 [15]. Additionally, frequent haemodialysis (6 times a week) led to better prognoses than conventional haemodialysis (3 times a week) in a prospective randomized study where eKt/V was significantly higher in conventional haemodialysis (1.41 ± 0.21) than frequent haemodialysis (1.06 ± 0.21) [16]. Our findings differ from some reports because we showed an adverse influence of high dose dialysis on the cardiovascular system. Despite the limited evidence about the causative relationship between high dose dialysis and VC, characteristics of our study subjects and haemodynamic changes caused by high dose dialysis may be an explanation for our findings. Our study subjects had a relatively long dialysis duration (81.5 ± 73.5 months). In these subjects, high dose dialysis might have an adverse influence on vasculature. Increased dialysis dose is characterized by a high blood flow rate through relatively larger membrane surface areas and pore size. Thus, it is postulated that higher dialysis doses may result in larger haemodynamic changes, promoting the loss of calcification inhibitors.

HDF is a widely used haemodialysis approach and is effective for removing middle weight molecules. Our results showed that HDF is associated with advanced VC. The loss of calcification inhibitors, including fetuin-A, may be an explanation for the significant association between HDF and advanced VC. Fetuin-A is a glycoprotein synthesized in the liver and expressed in the extracellular space and known to be an inhibitor of VC in dialysis patients [17]. Dekker et al. recently compared serum calcification propensity between high-flux haemodialysis and HDF. In their analysis, HDF had a larger effect on the change in fetuin-A concentrations as compared to HD (*p* = 0.002), and the change of fetuin-A concentration between pre- and post-dialysis was − 0.46% in the HD group and − 3.39% in the HDF group [18]. Their results suggest that increased removal of fetuin-A may be a potential mechanism for the significant association between HDF and advanced VC.

There were some limitations to this study. Unfortunately, our sample size was not sufficient to support the hypothesis for our findings. The major limitation of our study was the inability to determine the underlying mechanisms for these results, which is due to the limitations of cross-sectional studies that cannot identify causative relationships and controlling confounders. Moreover, it is plausible that patients with higher dialysis doses had higher levels of uremic toxin, leading to the significant association between higher dialysis dose and advanced VC. It has been demonstrated that the accumulation of uremic toxins, including inorganic phosphate, idoxyl-sulfate, and advanced glycation end-products is responsible for the high prevalence of vascular calcification in CKD patients [19]. However, evidence is still insufficient, and thus, further studies should be done to elucidate the potential mechanisms underlying the association between high dialysis dose and VC.

Hyponatremia is frequently manifested in CKD patients due to volume overload and diuretic medication. The results of our study showed that advanced VC was significantly associated with low sodium concentrations. Previous studies have reported that hyponatremia was associated with poor prognoses in dialysis patients, regardless of the severity of kidney disease [20–22]. Moreover, two recent studies indicated that a 4 mmol/L increase in baseline sodium was associated with 19–28% lower risk of all-cause mortality in haemodialysis patients [23, 24]. Interestingly, all our study groups had normal ranges of sodium concentration with an overall mean sodium concentration of 137.6 ± 3.4 mmol/L. This finding suggests that low sodium concentration even within the normal range contributes to the progression of VC.

Our study was based on the hypothesis that VC progresses because of interactions between multiple factors. The factors include classic cardiovascular risk factors, and other clinical conditions, accompanied by haemodialysis. Our study indicates that multiple clinical and haemodialysis parameters are involved in the progression of VC, which may provide additional insight about clinical conditions that could predispose patients to VC. Nonetheless, our results should be viewed within the perspective of its cross-sectional design. It is known that cross-sectional studies are limited as this approach cannot identify causative relationships or control for potential confounders. Thus, prospective studies are necessary to identify the longitudinal relationship between high dose dialysis and advanced VC. Additionally, specific mechanisms for our findings are not supported by our data. Although we suggested that haemodynamic change and over-clearance of fetuin-A by high dose dialysis could be a potential mechanism for our findings, we were not able to use appropriate laboratory evidence for our hypothesis. Moreover, there is a possibility that higher uremic toxins in subjects with higher dialysis dosing contribute to the significant association between high dialysis dose and advanced VC. Further studies should be conducted to elucidate the mechanism for the association between high dose dialysis and advanced VC.

## Conclusion

The results of our study indicated that Kt/V categories and HDF are significantly associated with advanced VC. This suggests that high dose dialysis may have an adverse impact on VC. However, our data are limited and we were not able to determine the causative relationship between high dose dialysis and VC. Further studies should be conducted to elucidate the underlying mechanisms for the association between high dose dialysis and VC.

## Supplementary information

**Additional file 1: Supplementary Table S1.** Odds ratio (95% CI) for advanced vascular calcification with adjustment for covariates including Charlson’s comorbidity index and vintage of dialysis in each haemodialysis parameter.

## Data Availability

The data of this study is based on K-cohort. K-cohort is in progress and is aim for finding risk factors related to cardiovascular complication in haemodialysis patients from 6 haemodialysis centers in Korea. We conducted this cross-sectional study by analyzing the baseline data of K-cohort.

## Abbreviations

BMI: Body mass index
CI: Confidence interval
CKD: Chronic kidney disease
CVD: Cardiovascular disease
DM: Diabetes mellitus
ESRD: End-stage renal disease
MAMC: Mid-arm mass circumference
MHD: Maintenance haemodialysis
OR: Odd ratio
VC: Vascular calcification
