# Supplementary material for: High dose haemodialysis and haemodiafiltration parameters and the relationship with advanced vascular calcification
Source: BMC Nephrol. 2020 Mar 6;21:86. doi: 10.1186/s12882-020-01738-4 (PMC7060583; doi:10.1186/s12882-020-01738-4)
Supplement: Supplementary file 1 — Additional file 1: Supplementary Table S1. Odds ratio (95% CI) for advanced vascular calcification with adjustment for covariates including Charlson’s comorbidity index and vintage of dialysis in each haemodialysis parameter. [file 12882_2020_1738_MOESM1_ESM.docx]

**Supplementary table 1.** Odds ratio (95% CI) for advanced vascular calcification with adjustment for covariates including Charlson’s comorbidity index and vintage of dialysis in each haemodialysis parameter

|  | Unadjusted model | Model 1^a^ | Model 2^b^ | Model 3^c^ |
| --- | --- | --- | --- | --- |
|  | OR (95% CI) | OR (95% CI) | OR (95% CI) | OR (95% CI) |
| DM | 1.99 (1.24-3.17) | 1.88 (1.10-3.17) | 1.94 (1.14-3.29) | 1.05 (1.03-1.08) |
| CAD | 2.93 (1.61-5.34) | 2.64 (1.36-5.14) | 2.78 (1.41-5.51) | 2.71 (1.39-5.29) |
| Single pool Kt/V | 6.5 (2.80-15.3) | 5.32 (1.56-18.14) | 5.27 (1.51-18.41) | 3.09 (2.47-3.51) |
| Equilibrated Kt/V | 8.8 (3.20-24.20) | 6.33 (1.53-26.17) | 6.16 (1.45-26.10) | 3.42 (2.61-4.14) |
| Standard Kt/V | 18.2 (4.90-68.20) | 10.91 (1.83-65.21) | 10.67 (1.74-65.52) | 6.21 (4.93-7.63) |
| HDF (reference HD) | 2.66 (1.51-4.68) | 3.28 (1.76-6.12) | 3.27 (1.74-6.16) | 2.35 (1.94-2.94) |
| Sodium | 0.85 (0.79-0.92) | 0.88 (0.81-0.96) | 0.88 (0.81-0.96) | 0.92 (0.85-0.98) |
| Chloride | 0.87 (0.83-0.93) | 0.89 (0.83-0.95) | 0.90 (0.83-0.96) | 0.93 (0.87-0.99) |
| Corrected Ca | 1.73 (1.25-2.40) | 1.60 (1.12-2.27) | 1.70 (1.17-2.46) | 1.53 (1.07-2.17) |

OR: odds ratio, CI: confidence interval, DM: diabetes mellitus, CAD: coronary artery disease, HD: Haemodialysis, HDF: haemodiafiltration, Ca: calcium

^a^ Adjusted for age, sex, DM, CAD, and dry BW

^b^ Adjusted for use of warfarin, dialysate calcium, and serum phosphate

^c^ Adjusted for Charlson’s comorbidity index, vintage of dialysis (months)
